# Supplementary material for: Alleles of a gene differ in pleiotropy, often mediated through currency metabolite production, in E. coli and yeast metabolic simulations
Source: Sci Rep. 2018 Nov 22;8:17252. doi: 10.1038/s41598-018-35092-1 (PMC6250661; doi:10.1038/s41598-018-35092-1)
Supplement: Supplementary file 1 — Supplementary Information [file 41598_2018_35092_MOESM1_ESM.pdf]

Supplementary Information:

Alleles of a gene differ in pleiotropy, often mediated through currency metabolite production, in *E. coli* and yeast metabolic simulations

Deya Alzoubi, Abdelmoneim Amer Desouki, Martin J. Lercher

Contents

Supplementary Figures 2

Supplementary Tables 11

Supplementary Figures

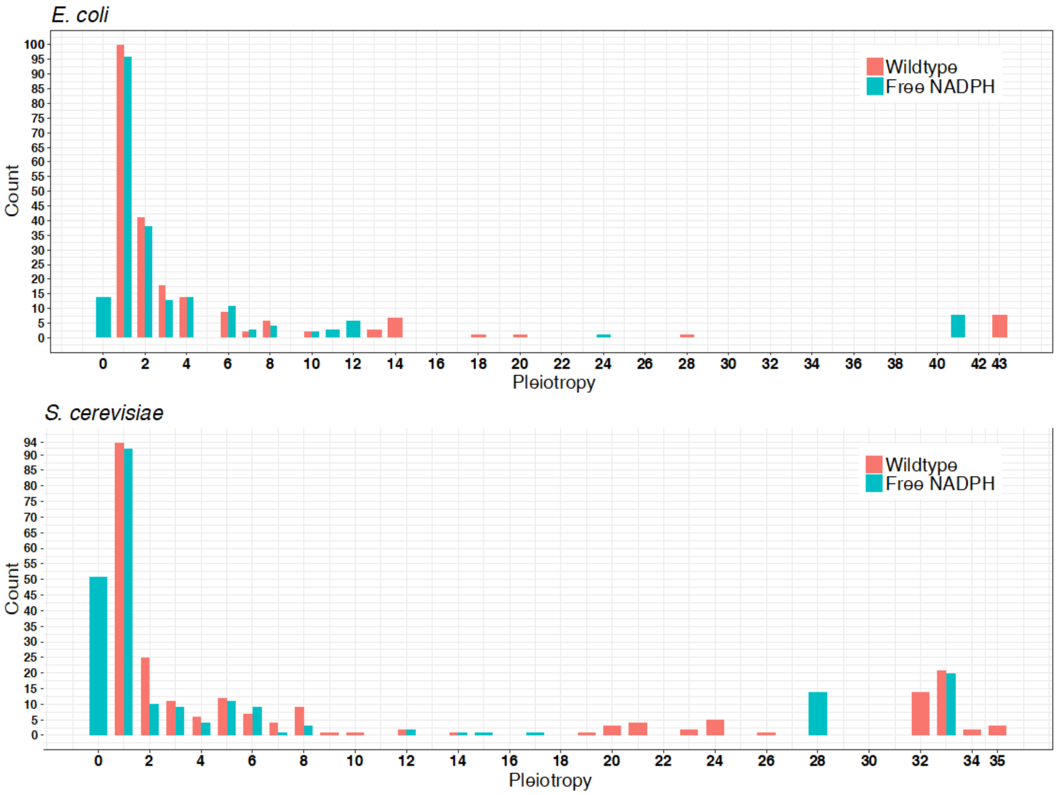

Figure S1: Distribution of the number of biomass components for which genes are essential even after allowing the mutant strain to adapt its protein expression (excluding genes with no effect on biomass production). The number of biomass components for which a given gene is essential is often reduced when NADPH is made freely available (cyan bars).

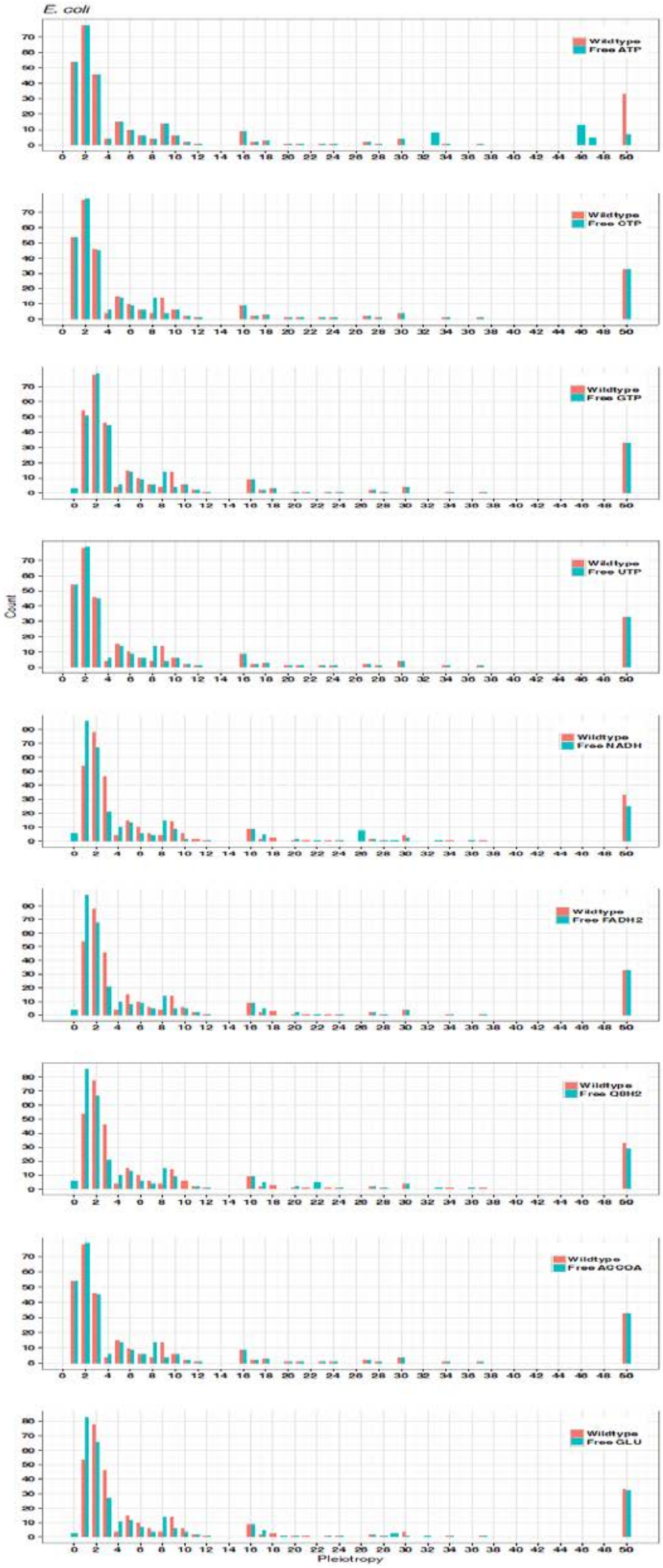
E. coli
Wildtype
Free ATP
Wildtype
Free CTP
Wildtype
Free GTP
Wildtype
Free UTP
Count
Wildtype
Free NADH
Wildtype
Free FADH2
Wildtype
Free Q8H2
Wildtype
Free ACCOA
Wildtype
Free GLU
Pleiotropy

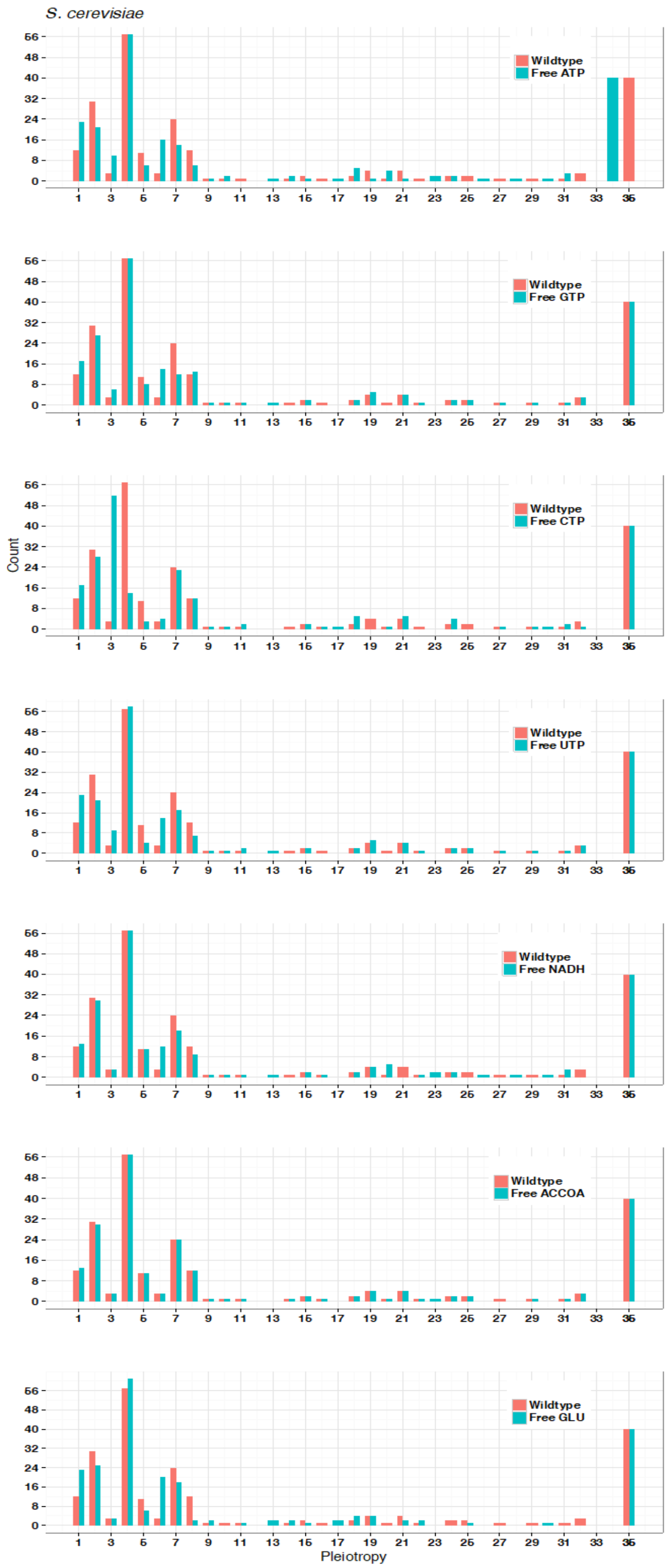

Figure S2. Distribution of pleiotropy for full gene knockouts. Pleiotropy is reduced for many *E. coli* and *S. cerevisiae* genes when different currency metabolites (one per panel) are made freely available.

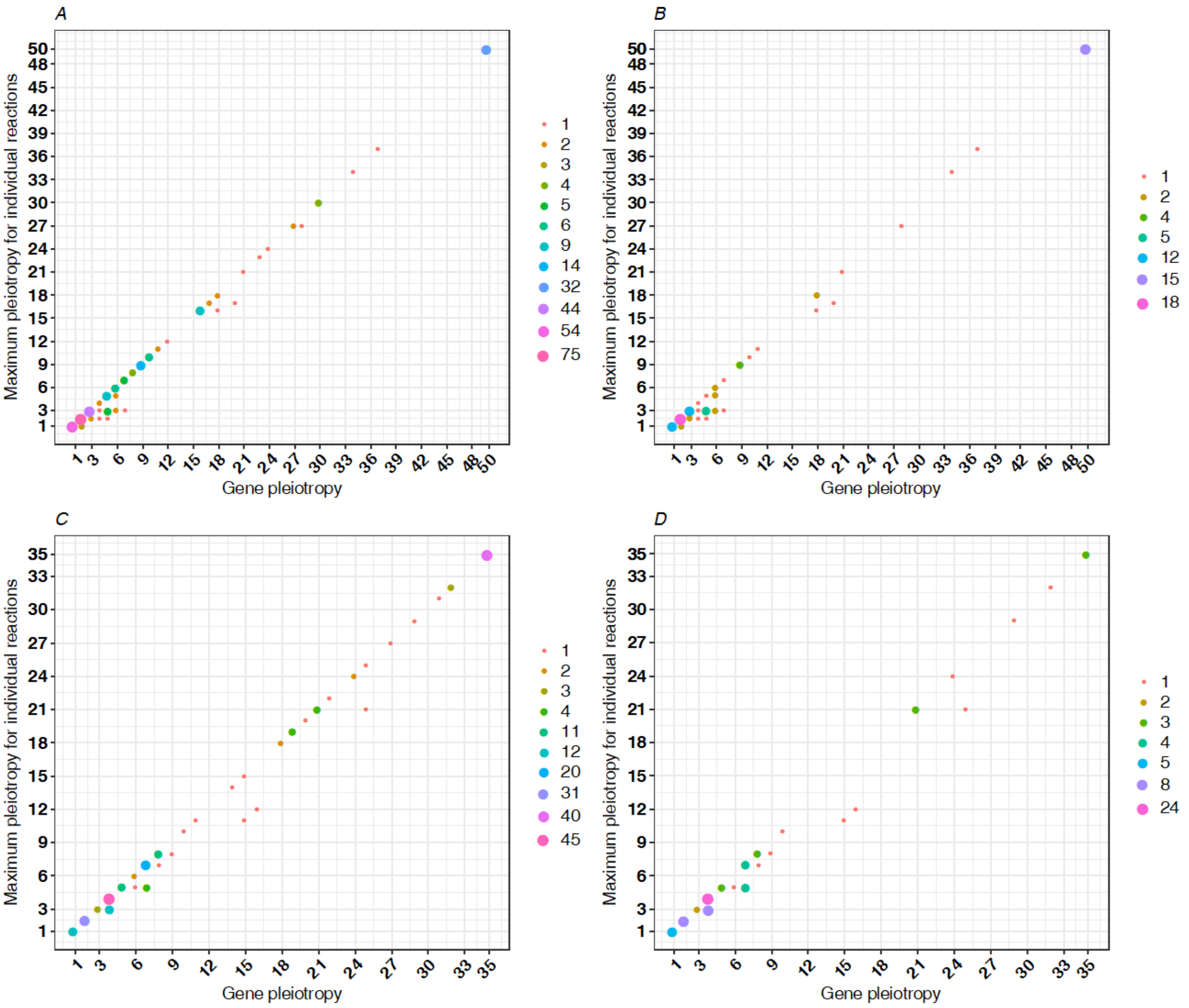

Figure S3. Comparison of gene pleiotropy (the number of biomass components affected by a full gene knockout) to the maximal pleiotropy observed when blocking the individual reactions for which this gene is essential. (A) *E. coli*, all genes; (B) *E. coli*, only genes essential for ≥2 reactions; (C) *S. cerevisiae*, all genes; (D) *S. cerevisiae*, only genes essential for ≥2 reactions. Dot size and color indicates the number of genes represented by each dot.

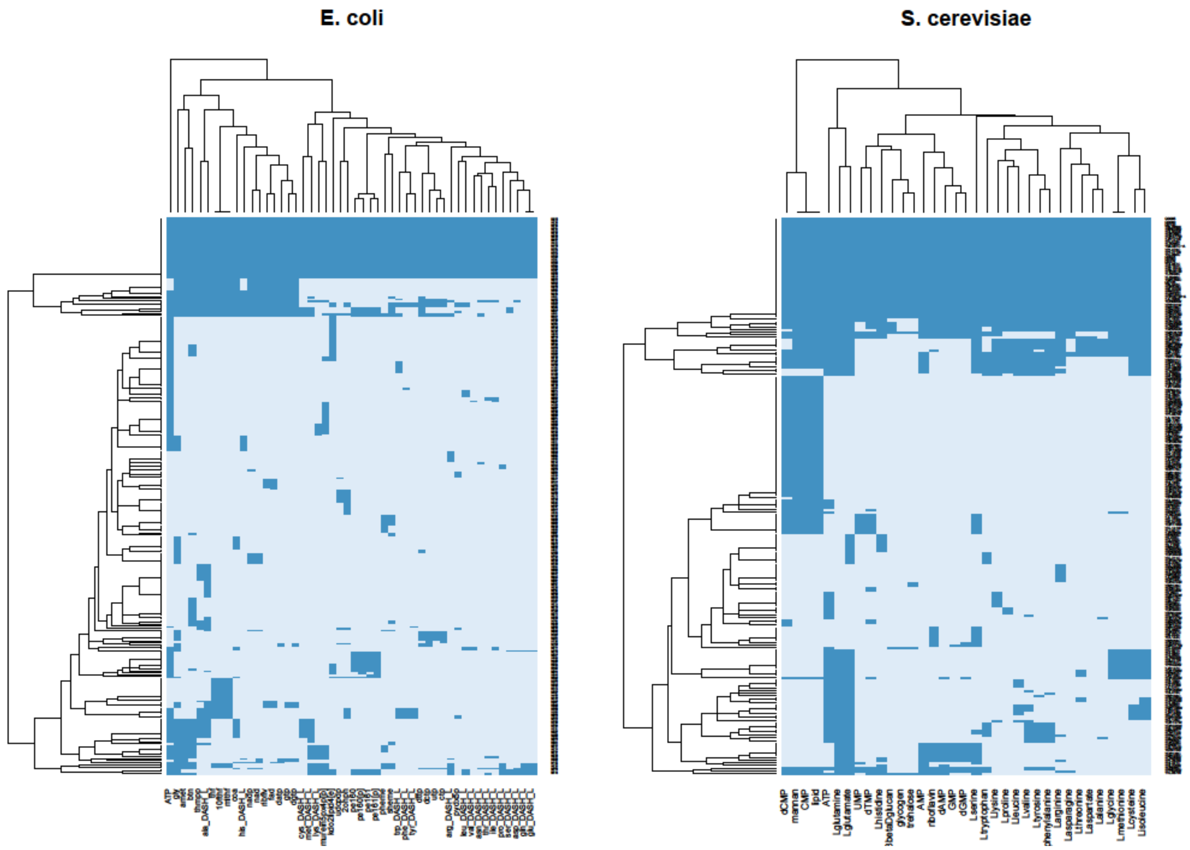

Figure S4. Graphical representation of the modularity of the bipartite pleiotropy networks for *E. coli* and *S. cerevisiae*. The figures show heatmaps produced with the R function of the same name, which order biomass components (columns) and genes (rows) through hierarchical clustering. Dark blue means that the biomass component's production is decreased through a knockout of the gene, light blue means it is not. Modularity shows up as blocks of dark blue, where several rows of gene affect the same neighbouring columns of traits.

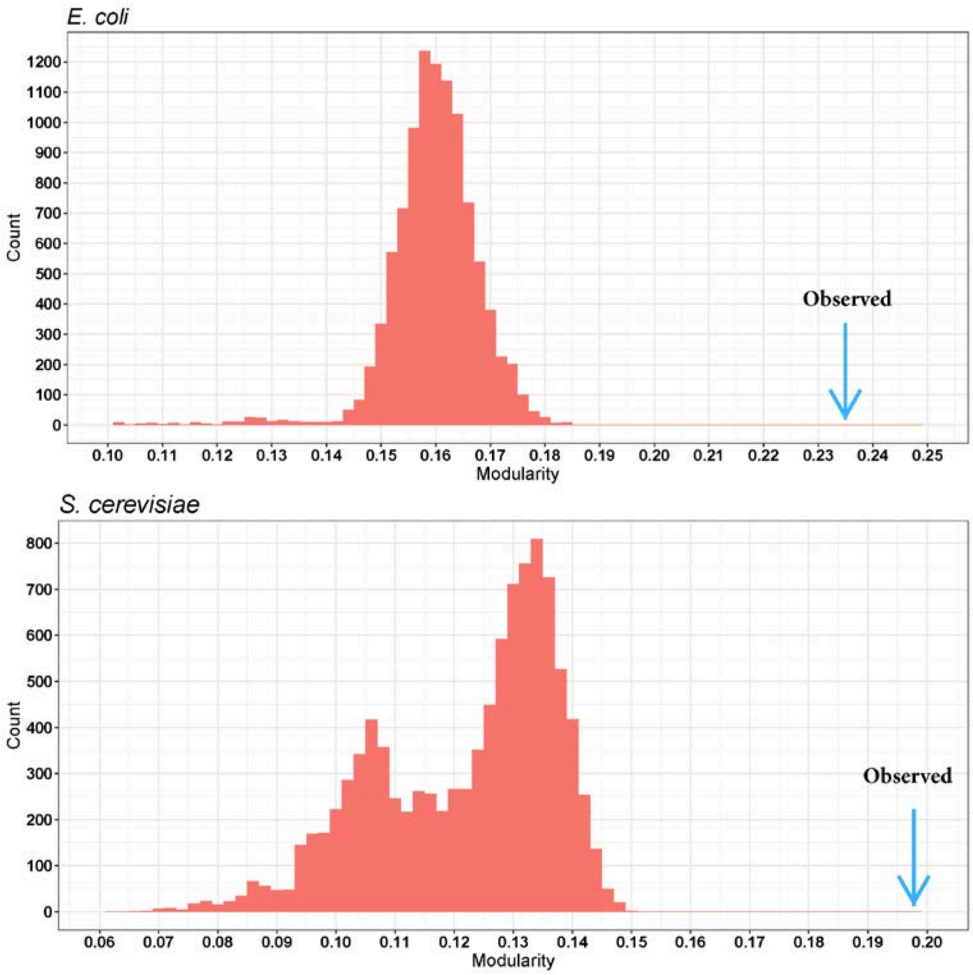

Figure S5. Observed modularity *Q* (as calculated with LP&BRIM, blue arrow) compared to the distribution of modularities obtained for pleiotropy networks with randomized links between genes and biomass components (red).

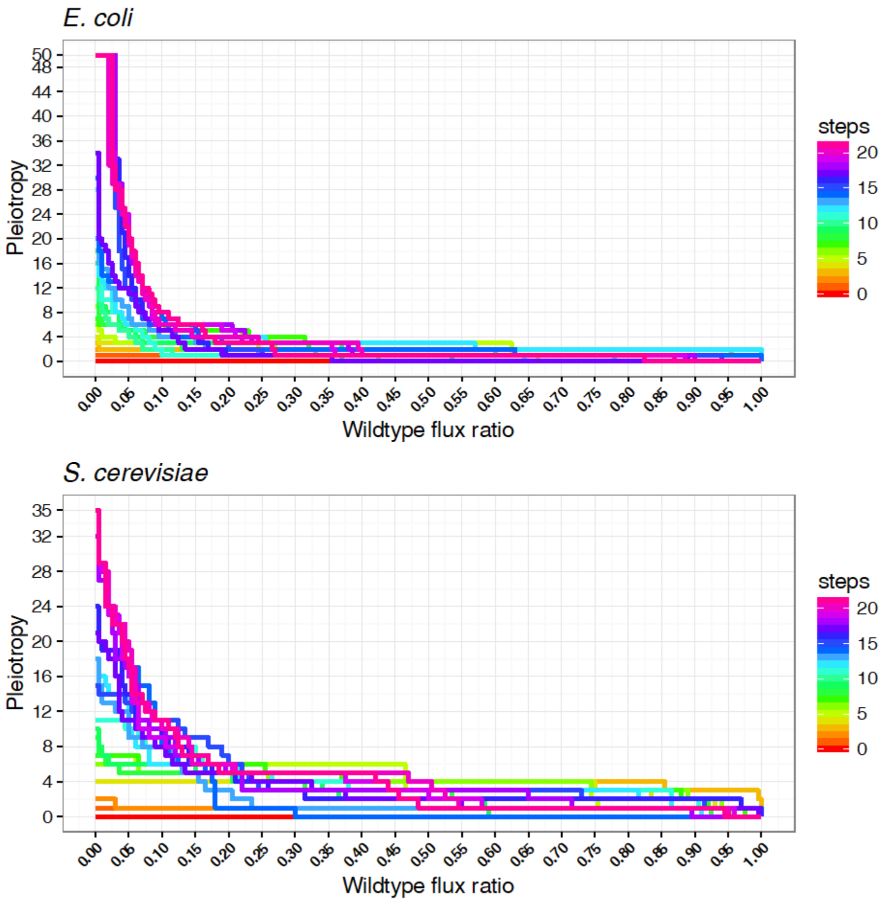

Figure S6. The pleiotropy of genes contributing to biomass production typically increases for increasingly debilitating mutations. Shown are curves for one randomly chosen gene for each value of the number of pleiotropy increases (steps).

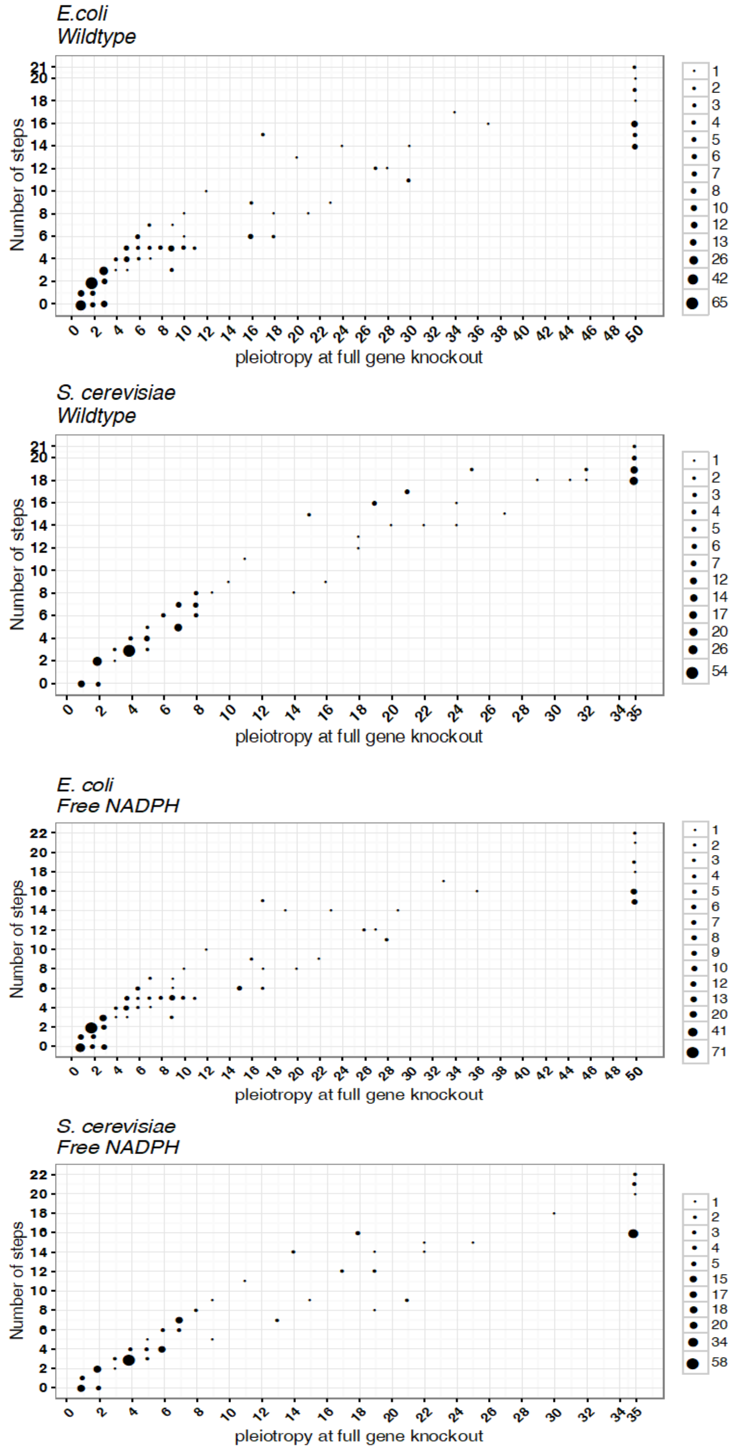

Figure S7. The number of step-wise increases in pleiotropy for increasingly debilitating mutations and the pleiotropy at full gene knockout are strongly correlated (Spearman's rank correlation when considering only genes contributing to biomass production, wildtype: ρ=0.926 (*E. coli*) and ρ=0.986 (*S. cerevisiae*); when making NADPH freely available: ρ=0.922 (*E. coli*) and ρ=0.986 (*S. cerevisiae*)). The diameter of each point is proportional to the number of genes with this combination of pleiotropy and step number.

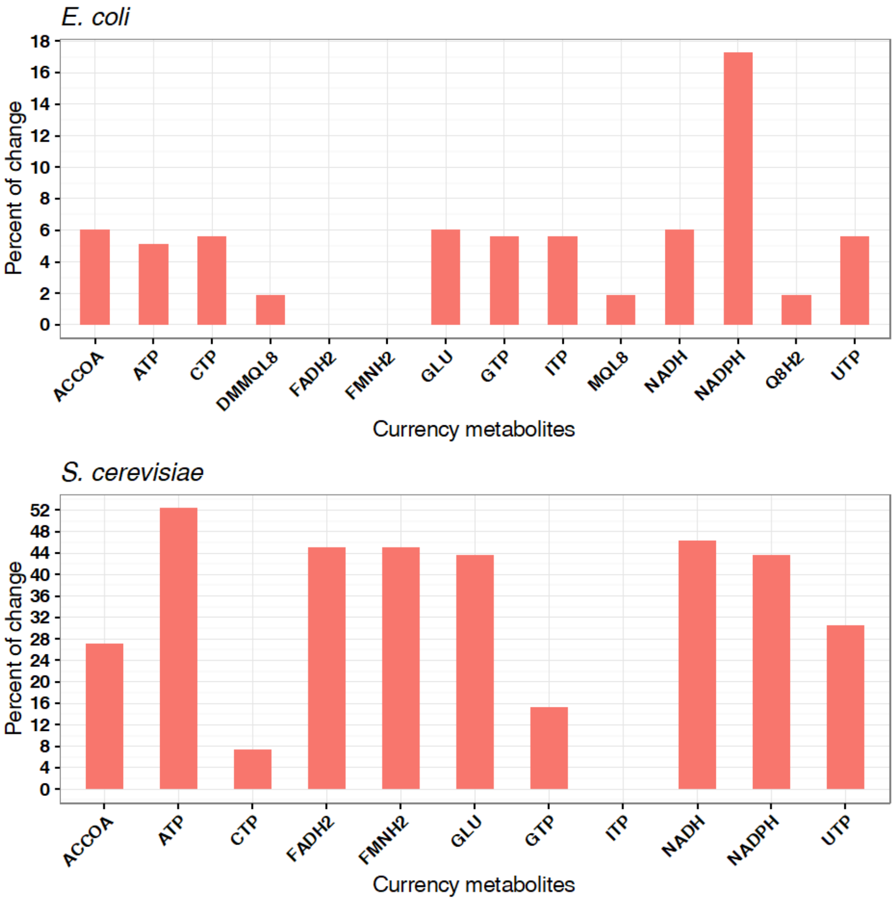

Figure S8. Percentage of genes for which free availability of a given currency metabolite reduces the number of biomass components for which this gene is essential even after allowing the mutant strain to adapt its protein expression to the altered gene content of its genome. Abbreviations: Adenosine triphosphate (ATP); Cytidine triphosphate (CTP); Guanosine triphosphate (GTP); Uridine triphosphate (UTP); Inosine triphosphate (ITP); Nicotinamide adenine dinucleotide (NADH); Nicotinamide adenine dinucleotide phosphate (NADPH); Flavin adenine dinucleotide reduced (FADH2); Reduced flavin mononucleotide (FMNH2); Ubiquinol-8 (Q8H2); Menaquinol 8 (MQL8); 2-Demethylmenaquinol 8 (DMMQL8); Acetyl-CoA (ACCOA); L-Glutamate (GLU).

Supplementary Tables

Table S1. Essential Biomass components in *E. coli* (excluding inorganic ions, H_2O, and products of the biomass reaction)

Components	
ala_DASH_L[c]	gtp[c]
arg_DASH_L[c]	utp[c]
asn_DASH_L[c]	murein5px4p[p]
asp_DASH_L[c]	kdo2lipid4[e]
cys_DASH_L[c]	pe160[c]
gln_DASH_L[c]	pe160[p]
glu_DASH_L[c]	pe161[c]
gly[c]	pe161[p]
his_DASH_L[c]	10fthf[c]
ile_DASH_L[c]	2ohph[c]
leu_DASH_L[c]	amet[c]
lys_DASH_L[c]	btn[c]
met_DASH_L[c]	coa[c]
phe_DASH_L[c]	fad[c]
pro_DASH_L[c]	mlthf[c]
ser_DASH_L[c]	nad[c]
thr_DASH_L[c]	nadp[c]
trp_DASH_L[c]	pheme[c]
tyr_DASH_L[c]	pydx5p[c]
val_DASH_L[c]	ribflv[c]
datp[c]	sheme[c]
dctp[c]	thf[c]
dgtp[c]	thmpp[c]
dttp[c]	udcpdp[c]
ctp[c]	atp[c]

Table S2. Essential Biomass components in *S. cerevisiae* (excluding inorganic ions, H_2O, and products of the biomass reaction)

Components	
ATP [cytoplasm]	L-proline [cytoplasm]
(1->3)-beta-D-glucan [illegible]	L-phenylalanine [cytoplasm]
lipid [cytoplasm]	L-tyrosine [cytoplasm]
mannan [cytoplasm]	L-histidine [cytoplasm]
glycogen [cytoplasm]	UMP [cytoplasm]
L-alanine [cytoplasm]	AMP [cytoplasm]
L-glycine [cytoplasm]	GMP [cytoplasm]
L-glutamate [cytoplasm]	CMP [cytoplasm]
L-glutamine [cytoplasm]	L-methionine [cytoplasm]
L-valine [cytoplasm]	L-cysteine [cytoplasm]
L-serine [cytoplasm]	L-tryptophan [cytoplasm]
L-leucine [cytoplasm]	trehalose [cytoplasm]
L-lysine [cytoplasm]	dAMP [cytoplasm]
L-threonine [cytoplasm]	dTMP [cytoplasm]
L-asparagine [cytoplasm]	dCMP [cytoplasm]
L-aspartate [cytoplasm]	dGMP [cytoplasm]
L-isoleucine [cytoplasm]	riboflavin [cytoplasm]
L-arginine [cytoplasm]	

Table S3. Currency metabolites and the corresponding supply reactions

Name	Abbrev.	Chemical equation	*E. coli* model equation	#Reactions in *E. coli*	*S. cerevisiae* model equation	#Reactions in *S. cerevisiae*
Adenosine triphosphate	ATP	ATP + H2O --> ADP + H(+) + Phosphate	atp[c] + h2o[c] --> adp[c] + h(+)[c] + pi[c]	359	0434 +0803 --> 0394 + 0794 + 1322	158
Cytidine triphosphate	CTP	CTP + H2O --> CDP + H(+) + Phosphate	ctp[c] + h2o[c] --> cdp[c] + h(+)[c] + pi[c]	18	0539 +0803 --> 0467 + 0794 + 1322	13
Guanosine triphosphate	GTP	GTP + H2O --> GDP + H(+) + Phosphate	gtp[c] + h2o[c] --> gdp[c] + h(+)[c] + pi[c]	25	0785 + 0803 --> 0739 +0794 + 1322	13
Uridine triphosphate	UTP	UTP + H2O --> UDP + H(+) + Phosphate	utp[c] + h2o[c] --> udp[c] + h(+)[c] + pi[c]	8	1559 + 0803 --> 1538 + 0794 + 1322	11
Inosine triphosphate	ITP	ITP + H2O --> IDP + H(+) + Phosphate	itp[c] + h2o[c] --> idp[c] + h(+)[c] + pi[c]	4	0950 + 0803 --> 0846 + 0794 + 1322	3
Nicotinamide adenine dinucleotide	NADH	Nicotinamide adenine dinucleotide - reduced --> H(+) + Nicotinamide adenine dinucleotide	nadh[c] --> h(+)[c] + nad[c]	119	1203 --> 0794 + 1198	36
Nicotinamide adenine dinucleotide phosphate	NADPH	Nicotinamide adenine dinucleotide phosphate – reduced --> H(+) + Nicotinamide adenine dinucleotide phosphate	nadph[c] --> h(+)[c] + nadp[c]	97	1212 --> 0794 + 1207	58
Flavin adenine dinucleotide reduced	FADH2	Flavin adenine dinucleotide reduced --> 2 H(+) + Flavin adenine dinucleotide oxidized	fadh2[c] --> 2 h(+)[c] +fad[c]	25	0689 --> 2 (0794) + 0687	2
Reduced flavin mononucleotide	FMNH2	Reduced FMN --> 2 H(+) + FMN	fmnh2[c] --> 2 h(+)[c] + fmn[c]	13	0717 --> 2 (0794) + 0714	3
Ubiquinol-8	Q8H2	Ubiquinol-8 --> 2 H(+) + Ubiquinone-8	q8h2[c] --> 2 h(+)[c] + q8[c]	24	NA	NA
Menaquinol 8	MQL8	Menaquinol 8 --> 2 H(+) + Menaquinone 8	mql8[c] --> 2 h(+)[c] + mqn8[c]	25	NA	NA
2-Demethylmenaquinol 8	DMMQL8	2-Demethylmenaquinol 8 --> 2 H(+) + 2-Demethylmenaquinone 8	2dmmql8[c] --> 2 h(+)[c] + 2dmmq8[c]	14	NA	NA
Acetyl-CoA	ACCOA	H2O + Acetyl-CoA --> H(+) + Acetate + Coenzyme A	h2o[c] + accoa[c] --> h(+)[c] + ac[c] + coa[c]	37	0803 + 0373 --> 0794 + 0362 + 0529	44
L-Glutamate	GLU	L-Glutamate + H2O --> 2-Oxoglutarate + Ammonium + 2 H(+)	glu_dash_l[c] + h2o[c] --> akg[c] +nh4[c] + 2h(+)[c]	49	0991 + 0803 --> 0180 + 0419 + 2 (0794)	44
